# A New Approach to Ultra‐Low Anterior Resection—Intersphincteric Dissection With Total Hiatal Ligament Excision for Very Low Rectal Cancer Located in the Posterior Wall of the Rectum: A More Satisfactory Technique for Local Recurrence Control

**DOI:** 10.1002/cam4.70307

**Published:** 2024-10-10

**Authors:** Shoufeng Li, Ye Wang, Huajun Cai, Zhen Pan, Xing Liu, Jinfu Zhuang, Guoxian Guan

**Affiliations:** ^1^ Department of Colorectal Surgery The First Affiliated Hospital of Fujian Medical University Fuzhou China; ^2^ Department of Colorectal Surgery National Regional Medical Center, Binhai Campus of the First Affiliated Hospital Fuzhou China

**Keywords:** circumferential tumor location, local recurrence, low rectal cancer, total hiatal ligament excision

## Abstract

**Background:**

The hiatal ligament, as the anatomical landmark for the completion of total mesorectal excision (TME) for ultra‐low rectal cancer, represents a continuation of the longitudinal muscle of the rectum. It receives vascular supply from the median sacral artery and contains lymphatic vessels. In cases where ultra‐low rectal cancer is located in the posterior rectal wall, the hiatal ligament may theoretically serve as an anatomical region susceptible to direct tumor cell spread or distant metastasis.

**Objective:**

To evaluate the effect of circumferential tumor location (CTL) on postoperative survival of low rectal cancer and to determine the effect of total hiatal ligament excision (THLE) on the prognosis of patients with posterior rectal cancer.

**Methods:**

Patients with ultra‐low rectal cancer who underwent laparoscopic surgery between March 2014 and October 2021 were enrolled in this study. Propensity score matching (PSM) analysis was used to compare the clinicopathological characteristics and prognosis of patients in the posterior group and the non‐posterior group. Prognostic factors were identified using COX regression. PSM analysis was also used in the posterior tumor subgroup to compare the clinicopathological characteristics and prognosis of patients in the hiatal ligament traditional transection (HLTT) and THLE groups.

**Results:**

After PSM, OS, and DFS were comparable between the posterior and non‐posterior groups. Similarly, no difference was noted in the local recurrence rate between the two groups (*p* = 0.23). The prognosis of ultra‐low rectal cancer was not affected by CTL. However, the local recurrence rate was significantly lower in the THLE group compared with the HLTT group (*p* = 0.023). Multivariate analysis of the posterior group identified CRM, TNM stage III, and HLTT as independent risk factors for local recurrence‐free survival.

**Conclusions:**

CTL is not a prognostic risk factor for low rectal cancer. In posterior wall tumors, THLE significantly reduces the local recurrence rate for low rectal cancer.

## 1 Introduction

Total mesorectal excision (TME) has proven to be the most significant advancement in rectal cancer treatment. Through the avascular embryologic plane, a precise and meticulous surgical procedure is performed to dissect the tumor, mesorectum, and associated lymph nodes [1]. Indeed, the introduction of the concept of anatomical dissection along embryologic planes has the potential to significantly contribute to the sustained and reproducible reduction in rectal cancer recurrence [2].

The hiatal ligament, composed of the longitudinal muscular layer of the rectum, serves as a crucial anatomical landmark for determining the termination point of ultra‐low anterior resection of the rectum in accordance with the principles of TME. This ligament is vascularized by the median sacral artery and contains lymphatic vessels [3]. The laparoscopic view displays the structure of the hiatal ligament as a two‐tined fork structure comprising a main trunk connected to the tip of the coccyx and two branches surrounding the posterior rectal wall, as previously illustrated in a video article published by our research group [4]. Theoretically, ultra‐low rectal cancer located in the posterior rectal wall may directly spread along the longitudinal muscle within the hiatal ligament or may metastasize distantly through vascular and lymphatic vessels present within the ligament. Our laparoscopic videos clearly delineate a distinct boundary between the hiatal ligament and the hiatus. In contrast to the traditional perception of a fibrous ligament or membrane, it appears as broad, robust, and significantly thick smooth muscle. To the best of our knowledge, this is the first study to present clear laparoscopic video footage demonstrating the completion of total hiatal ligament excision (THLE) [5]. We hypothesized that THLE improves local control and long‐term prognosis in patients with ultra‐low rectal cancer affecting the posterior rectal wall.

On the other hand, the asymmetry in the circumferential anatomical adjacency of ultra‐low rectal cancer, resulting from the attachment of the hiatal ligament to the posterior wall of the rectum rather than the anterior wall, may potentially lead to asymmetrical oncologic outcomes. While studies have investigated the prognosis of patients based on circumference, their results have been highly heterogeneous. Emsle et al. [6] reported that posterior tumors are associated with worse survival outcomes. Conversely, Chan et al. [7] and Wu et al. [8] demonstrated that posterior wall tumors had a lower recurrence rate and longer survival time. Garcıá‐Granero et al. [9] and Park et al. [10] described that posterior wall location was not associated with prognosis. However, these studies recruited not solely patients with ultra‐low rectal cancer but also those with middle rectal cancer. Furthermore, studies have not offered a comprehensive and detailed anatomical description, along with surgical management of the hiatal ligament.

The current study exclusively included patients with ultra‐low rectal cancer, potentially involving the hiatal ligament, to assess the effect of circumferential tumor location (CTL) on survival outcomes. To our knowledge, no studies have evaluated the effect of the hiatal ligament on postoperative tumor recurrence in patients with ultra‐low rectal cancer. Furthermore, a comparative analysis was carried out to examine the impact of traditional transection and total excision of the hiatal ligament on the prognosis of patients with ultra‐low rectal cancer located in the posterior rectal wall.

## 2 Materials and Methods

### 2.1 Patients

Our hospital consecutively diagnosed and treated 2659 patients with colorectal cancer from March 2014 to October 2021. This study was approved by the ethics committee of The First Affiliated Hospital of Fujian Medical University. Rectal cancer was staged according to the seventh edition of the TNM staging standard by the American Joint Committee on Cancer [11].

All surgeons in this study performed laparoscopic TME. The two approaches for resecting the hiatal ligament, namely, traditional transection and total excision of the ligament, were based on varying understandings among the surgeons in our team regarding the hiatal ligament. The two groups of doctors were from the same medical team and had similar surgical experience and technical level.

All patients were evaluated by preoperative staging workups. Following radical surgery, treatment using adjuvant therapy was determined according to ESMO guidelines [12].

Patients undergoing neoadjuvant therapy received 45 Gy/25 long‐course radiation therapy to the pelvis over 5 weeks, followed by 5.4 Gy boosts of radiation therapy to the primary tumor. Both capcitabine and oxaliplatin (CapeOX) and 5FU and oxaliplatin (FOLFOX) were administered as preoperative chemotherapy regimens.

### 2.2 Inclusion and Exclusion Criteria

The inclusion criteria were: (1) histologically confirmed rectal adenocarcinoma; (2) tumors located ≤ 5 cm above the anal edge; (3) transrectal ultrasound (ERUS) and magnetic resonance imaging (MRI) displayed that the tumor did not involve the external anal sphincter or peripheral structures; (4) preoperative assessment did not reveal distant metastases; and (5) patients with normal anal sphincter function (Wexner fecal continence score < 4).

The exclusion criteria were: (1) patients with synchronous cancers; (2) patients who underwent emergency surgery and palliative removal; (3) patients with stage T4 after preoperative chemoradiotherapy; and (4) circumferential tumors involving the whole 360° circumference of the rectal wall.

### 2.3 Propensity Score Matching

Depending on the location of the tumor, covariates such as gender, age, BMI, ASA score, tumor size, location of tumor from the anal verge, CEA and CA19‐9 levels, neoadjuvant therapy, postoperative adjuvant therapy, operation time, postoperative complications, degree of differentiation, TNM staging, and complete resection of the hiatal ligament were matched using 1:1 propensity score matching (PSM). The matching tolerance was set at 0.02, and no replacement was performed. In the entire cohort, matching resulted in 376 patients according to tumor orientation. Similarly, PSM was performed in the posterior tumor group according to hiatal ligament treatment using the same covariates and parameters. In the posterior tumor group, according to the processing of the hiatal ligament, 376 patients were included.

### 2.4 Circumferential Involvement

Surgical records were retrospectively reviewed to gather information on CTL. In our study, the rectal wall was divided into 3 sections, namely anterior, lateral, and posterior (Figure S1A). Back positions were represented by the posterior quadrant, which is located between 4 o'clock and 8 o'clock. Tumors involving the posterior quadrant were categorized as posterior wall rectal cancer, regardless of the involvement of other quadrants. (excluding circumferential tumors) (Figure S1B,C). Tumors that did not involve the posterior quadrant were considered non‐posterior wall tumors (Figure S1D).

### 2.5 Surgical Procedures

**FIGURE 1:**
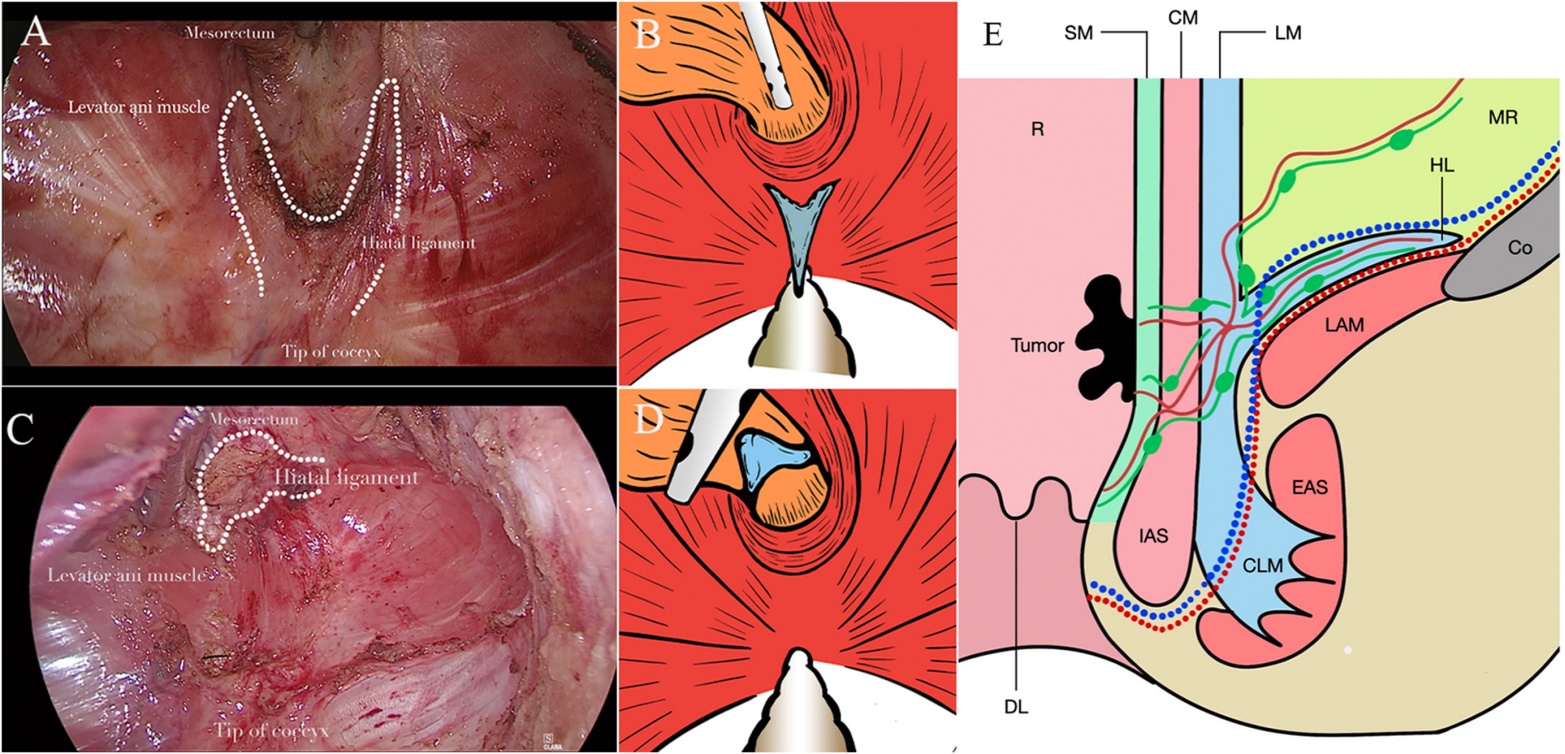
Intraoperative images and schematic representation of the total hiatal ligament excision group and the hiatal ligament traditional transection group. (A). Intraoperative images of the hiatal ligament traditional transection group. (B). Schematic diagram of the hiatal ligament traditional transection group. (C). Intraoperative images of total hiatal ligament excision. (D). Schematic diagram of total hiatal ligament excision. (E). Sagittal section of the posterior wall in the hiatal ligament traditional transection group (blue dotted line) and total hiatal ligament excision group (red dotted line). CLM, circular longitudinal muscle; CM, circular muscle; Co, coccyx; DL, dentate line; EAS, external anal sphincter; HL, hiatal ligament; IAS, internal anal sphincter; LAM, levator ani muscle; LM, longitudinal muscle; MR, mesorectum; R, rectum; and SM, submucosa.

Intraoperative images of the two surgical methods are illustrated in Figure 1. Laparoscopic TME was performed according to Heald's TME principle [13]. Adequately identifying the boundaries of the hiatal ligament before proceeding with further manipulation may potentially simplify the surgical procedure.

Identification of the hiatal ligament: It is positioned within the coccygeal area of the levator ani hiatus, particularly posterior to the rectum and superior to the coccyx. The typical hiatal ligament exhibits a Y‐shaped structure comprising two branches and a main trunk. The posterior rectal wall (viewed laparoscopically, approximately from the 4 o'clock to 8 o'clock positions) is characterized by a distinct attachment of the hiatal ligament.

Resection technique: In HLTT, please refer to the 2023 Delphi consensus regarding the surgical technique for the hiatal ligament [14]. In THLE, after adequate rectal mobilization, the main trunk and both branches of the hiatal ligament were identified. The levator ani fascia was incised 1 cm on each side of the hiatal ligament, exposing the pubococcygeus muscle and the puborectalis muscle in order to completely resect the entire hiatal ligament. Electrocautery, electroscissors, and other monopolar energy platforms were employed for resecting the hiatal ligament. An ultrasonic scalpel was only utilized for hemostasis.

Verification of resection: After resection, the adequacy of the surgery was verified by inspecting the surgical field through the laparoscopic view to ensure the absence of residual smooth muscle tissue surrounding the pubococcygeus muscle, puborectal muscle, and levator hiatus. The fascia on the surface of the coccygeal tip and the levator ani fascia should appear continuous and smooth, without any remaining smooth muscle termini.

Documentation: The resection process, including the defined boundaries, resection technique, and verification after resection, was meticulously documented in the surgical record. This ensured accurate reporting and reproducibility of the method.

### 2.6 Follow‐Up

Imaging assessments, including chest CTs and abdominal pelvic MRIs, were performed every 3 months for the first 2 years and annually thereafter. After the initial surgical intervention, colonoscopies were performed 3 months–1 year later. Patients were followed up through telephone calls and outpatient and inpatient visits.

### 2.7 Endpoints

DFS was defined as the duration from diagnosis until recurrence, metastasis, or the last follow‐up censored in November 2022. LRFS was defined as the duration from diagnosis to local recurrence or the last follow‐up censored in November 2022. CRM involvement was considered when the tumor was within 1 mm of the excised specimen [15].

### 2.8 Statistical Analysis

Categorical variables were analyzed using the chi‐squared test or Fisher's exact test, whereas continuous variables were compared using the Student's *t*‐test or Mann–Whitney *U* test. Survival analysis was conducted using Kaplan–Meier analysis. Prognostic factors were analyzed using absolute and multivariate Cox proportional hazards regressions. Two‐sided *p* > 0.05 were considered statistically significant.

## 3 Results

### 3.1 Clinicopathological Characteristics

**FIGURE 2:**
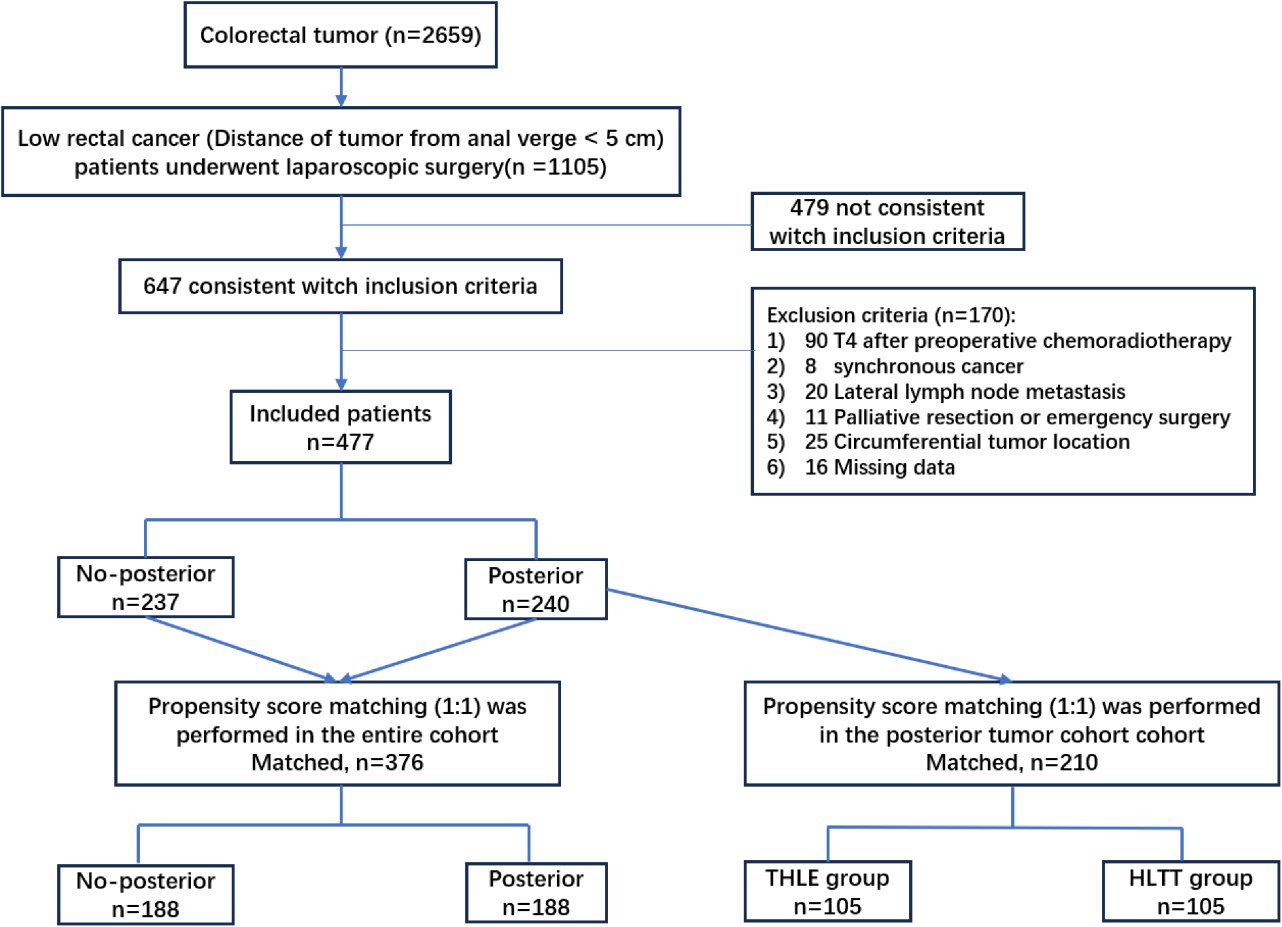
Patient selection diagram.

The flow chart of patient selection is displayed in Figure 2. A total of 477 patients were eventually enrolled in this study, with 240 (50.3%) patients in the posterior group and 237 (49.7%) patients in the non‐posterior group. Their clinicopathological characteristics are summarized in Table 1. The mean age of patients was 55.3 ± 10.6 years, with 282 (59.1%) men and 195 (40.9%) women. Age, gender, BMI, ASA, CEA, and CA19‐9 levels, tumor distance from the anal verge, and neoadjuvant therapy were comparable between the posterior and non‐posterior groups. After PSM, 188 patients were assigned to the posterior group and non‐posterior group, respectively, with no significant differences in tumor location and clinicopathological characteristics (*p* > 0.05).

**TABLE 1:** Baseline characteristics of the unmatched cohort and the propensity score‐matched adjusted cohort.

Variable	Unmatched cohort			PSM‐adjusted cohort		
	Posterior (*n* = 240)	No‐posterior (*n* = 237)	*p*	Posterior (*n* = 188)	No‐posterior (*n* = 188)	*p*
Sex, *n* (%)			0.363			0.833
Male	137 (57.1)	145 (61.2)		114 (60.6)	112 (59.6)	
Female	103 (42.9)	92 (38.8)		74 (39.4)	76 (40.4)	
Age (year) mean ± SD	54.8 ± 11.5	55.9 ± 9.3	0.115	55.8 ± 10.8	55.1 ± 9.4	0.453
BMI (kg/m^2^) ± SD	22.9 ± 4.8	22.8 ± 2.8	0.745	22.9 ± 5.1	22.6 ± 2.9	0.525
ASA score, *n* (%)			0.347			0.562
I/II	226 (94.2)	218 (92.0)		172 (91.5)	175 (93.1)	
III	14 (5.8)	19 (8.0)		16 (8.5)	13 (6.9)	
Distance from anal verge (cm), mean ± SD	4.3 ± 0.6	4.3 ± 0.6	0.192	3.8 ± 0.9	3.8 ± 0.8	0.292
Tumor size (cm), mean ± SD	2.7 ± 1.2	2.6 ± 1.0	0.206	2.7 ± 1.2	2.7 ± 0.9	0.674
CEA, ng/mL, *n* (%)			0.48			0.899
≤ 5	185 (79.7)	189 (79.7)		149 (79.3)	148 (78.7)	
> 5	55 (20.3)	48 (20.3)		39 (20.7)	40 (21.3)	
CA19‐9, ng/mL, *n* (%)			0.46			0.348
≤ 37	215 (89.6)	217 (91.6)		170 (90.4)	175 (93.1)	
> 37	25 (10.4)	20 (8.4)		18 (6.4)	13 (6.9)	
Neoadjuvant therapy, *n* (%)			0.057			0.711
Short‐course radiotherapy	2 (0.8)	1 (0.4)		2 (1.1)	1 (0.4)	
Chemotherapy	1 (0.4)	0 (0)		1 (0.5)	0 (0)	
Chemoradiotherapy	144 (60.0)	169 (71.3)		123 (65.4)	126 (67.0)	
None	93 (38.8)	67 (28.3)		61 (33.0)	61 (32.4)	
Operative time, min, mean ± SD	211.9 ± 50.5	212.3 ± 59.1	0.767	210.3 ± 49.0	214.4 ± 61.0	0.502
Blood loss, mL, median (range)	50 (10–400)	50 (5–400)	0.149	50 (10–400)	50 (10–200)	0.878
Postoperative complications, *n* (%)	64 (26.7)	70 (29.5)	0.486	49 (26.7)	49 (29.5)	1
Distal cutting margin (cm), mean ± SD	1.8 ± 0.6	1.8 ± 0.6	0.718	1.8 ± 0.6	1.8 ± 0.6	0.718
Pathologic T stage, *n* (%)			0.193			0.176
pT1	26 (10.8)	30 (12.7)		17 (9.0)	26 (13.8)	
pT2	85 (35.4)	97 (40.9)		61 (32.4)	73 (38.8)	
pT3	77 (32.1)	71 (30.0)		67 (35.6)	58 (30.9)	
pT4	1 (0.4)	4 (1.7)		1 (0.5)	2 (1.1)	
pT0	51 (21.3)	35 (14.8)		42 (22.3)	29 (15.4)	
Pathologic N stage, *n* (%)			0.996			0.969
pN0	171 (70.9)	168 (70.9)		134 (71.3)	134 (71.3)	
pN1	57 (24.1)	57 (24.1)		45 (23.9)	44 (23.4)	
pN2	12 (5.1)	12 (5.1)		9 (4.8)	10 (5.3)	
Pathologic TNM stage, *n* (%)			0.504			0.764
I	99 (41.3)	91 (38.4)		72 (38.3)	71 (37.8)	
II	44 (18.3)	38 (16.0)		38 (20.2)	32 (17.0)	
III	60 (25.0)	74 (31.2)		48 (25.5)	56 (29.8)	
pCR	37 (15.4)	34 (14.3)		30 (16.0)	29 (15.4)	
Histological differentiation, *n* (%)			0.181			0.904
Well	4 (1.7)	1 (0.4)		2 (1.7)	2 (1.1)	
Moderate	229 (95.4)	233 (98.3)		183 (97.3)	184 (97.9)	
Poor	7 (2.9)	3 (1.3)		3 (1.6)	2 (1.1)	
CRM, *n* (%)			0.984			1
Negative (> 1 mm)	235 (97.9)	232 (97.9)		184 (97.9)	184 (97.9)	
Positive (≤ 1 mm)	5 (2.1)	5 (2.1)		4 (2.1)	4 (2.1)	
Treatment of hiatal ligament						0.466
THLE	133 (55.4)	114 (48.1)		103 (58.5)	110 (58.5)	
HLTT	107 (44.6)	123 (51.9)		85 (45.2)	85 (41.5)	
Adjuvant therapy, *n*			0.61			0.939
Chemotherapy	172 (71.7)	179 (75.5)		136 (72.3)	139 (73.9)	
Chemoradiotherapy	3 (1.3)	2 (0.8)		2 (1.1)	2 (1.1)	
None	65 (27.1)	56 (23.6)		50 (26.6)	47 (25.0)	

Abbreviations: CRM, circumferential resection margin (tumor ≤ 1 mm from the margin); HLTT, hiatal ligament traditional transection group; pCR, pathological complete response; SD, standard deviation; THLE, total hiatal ligament excision.

### 3.2 Comparison of Perioperative Outcomes

The perioperative results are presented in Table 1. There were no significant differences in operative time, postoperative complications, distal cutting margin, and blood loss between the posterior and non‐posterior groups.

### 3.3 Oncological Outcomes

**FIGURE 3:**
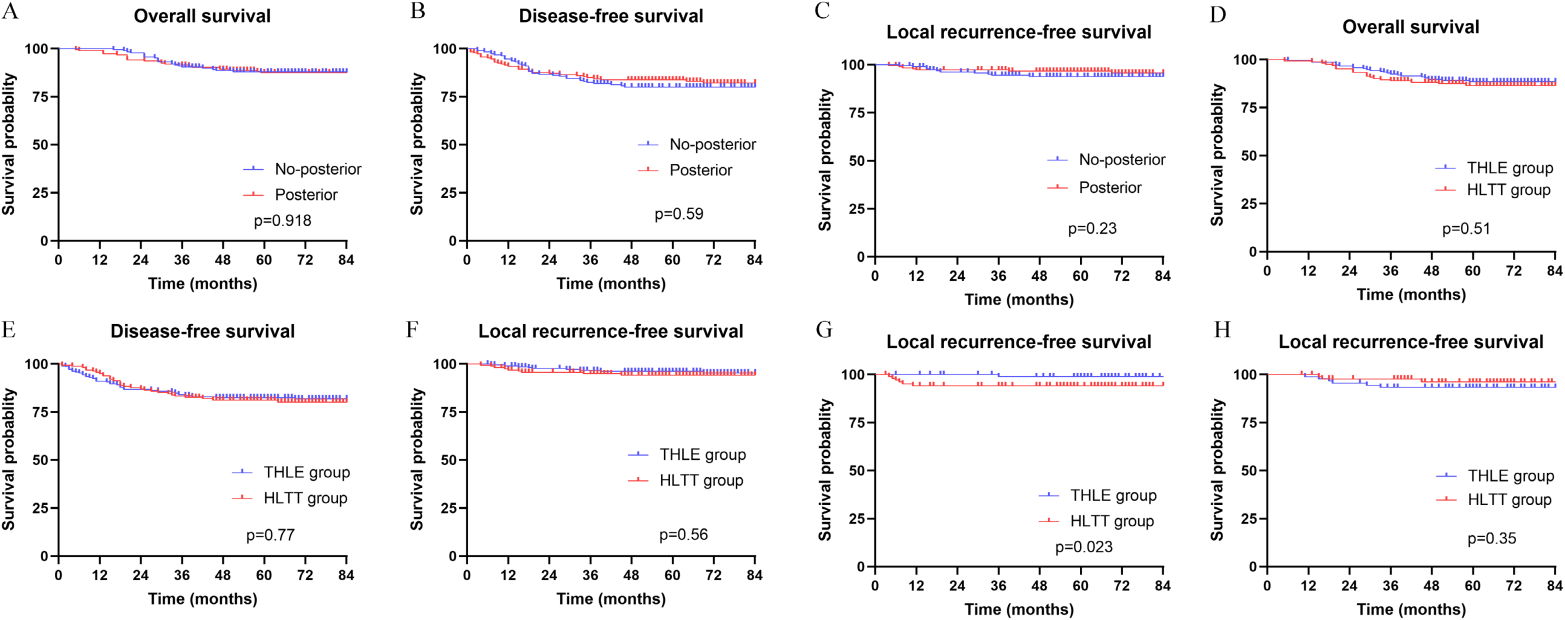
(A–C) Kaplan–Meier curves depicting overall survival, disease‐free survival, and local recurrence according to circumferential tumor location in the propensity score matching‐adjusted cohort. (D–F) Kaplan–Meier curves illustrating overall survival, disease‐free survival, and local recurrence according to the treatment of hiatal ligament in the propensity score matching‐adjusted cohort. (G–H) Kaplan–Meier curve presenting local recurrence rates in the posterior and non‐posterior groups in the propensity score matching‐adjusted cohort. G. posterior group. H. no‐posterior group.

The median follow‐up time was 72 months (range 3–128 months). Figure 3 delineates Kaplan–Meier curves for OS, DFS, and LRFS. After PSM, there was no significant difference in OS and DFS among the posterior and non‐posterior groups (5‐year OS: 87.4% vs. 87.9%, *p* = 0.918; 5‐year DFS: 83.4% vs. 80.1%, *p* = 0.59). Likewise, no significant difference was noted in the local recurrence rate between the posterior and non‐posterior groups (5‐year: 4.26% vs. 6.38%, *p* = 0.359). Finally, no significant difference was detected in the local recurrence rate between the HLTT group and the THLE group (5‐year: 6.13% vs. 4.69%, *p* = 0.539).

### 3.4 Analysis of Prognostic Factors in the Entire Cohort

The results of the univariate analysis for OS, DFS, and LRFS are presented in Tables S1 and S2. As anticipated, variables such as TNM stage III (*p* = 0.001) and CRM status (*p* < 0.001) significantly influenced OS. Meanwhile, CEA level (DFS: *p* = 0.013; LRFS: *p* = 0.029), TNM stage III (DFS: *p* < 0.001; LRFS: *p* = 0.002), and CRM status (DFS: *p* < 0.001; LRFS: *p* < 0.001) significantly affected DFS and LRFS. However, there was no significant difference in outcomes between the THLE and HLTT groups (Figure 3).

In multivariate analysis, the significant factors predicting OS, DFS, and LRFS were pathologic TNM stage III (OS: HR, 3.200, *p* = 0.004; HR; DFS: HR, 2.902, *p* = 0.001; LRFS: HR, 7.786, *p* = 0.008) and CRM status (OS: HR, 5.222, *p* < 0.001; DFS: HR, 2.957, *p* = 0.014; LRFS: HR, 4.968, *p* = 0.014, Table 2).

**TABLE 2:** Multivariate analysis of overall survival, disease‐free survival, and local recurrence‐free survival in the post‐matching cohort.

Variable	Multivariate analysis	
	HR (95% CI)	*p*
Overall survival		
CRM		
Negative	1 (reference)	
Positive	5.222 (2.140–12.738)	**< 0.001**
Pathologic TNM stage		
Stage I	1 (reference)	
Stage II	2.006 (0.796–5.057)	0.140
Stage III	3.200 (1.456–7.034)	**0.004**
pCR	1.129 (0.347–3.667)	0.840
Disease‐free survival		
CEA, ng/mL, *n* (%)		
≤ 5	1 (reference)	
> 5	1.555 (0.925–2.614)	0.095
CRM		
Negative	1 (reference)	
Positive	2.957 (1.242–7.038)	**0.014**
Pathologic TNM stage		
Stage I	1 (reference)	
Stage II	1.241 (0.563–2.737)	0.593
Stage III	2.902 (1.579–5.334)	**0.001**
pCR	1.241 (0.563–2.737)	0.489
Local recurrence‐free survival		
CEA, ng/mL, *n* (%)		
≤ 5	1 (reference)	
> 5	1.32 (0.731–4.591)	0.197
CRM		
Negative	1 (reference)	
Positive	4.968 (1.383–17.845)	**0.014**
Pathologic TNM stage		
Stage I	1 (reference)	
Stage II	1.930 (0.271–13.736)	0.511
Stage III	7.786 (1.708–35.489)	**0.008**
pCR	2.396 (0.337–17.017)	0.382

*Note:* Bold values indicate a *p* value < 0.05, which is statistically significant.

Abbreviations: CRM, circumferential resection margin (tumor ≤ 1 mm from the margin); pCR, pathological complete response.

### 3.5 Subgroup Analysis

After PSM, age, gender, BMI, ASA score, CEA and CA19‐9 levels, tumor distance from the anal verge, and the proportion of patients receiving neoadjuvant therapy were comparable between THLE and HLTT groups. After PSM, there were 105 patients in both the THLE and HLTT groups, with no significant differences in tumor location and clinicopathological characteristics (*p* > 0.05) (Table S3). Furthermore, LRFS outcomes were analyzed using CTL subgroups. The posterior group was stratified into two subgroups, namely the THLE and HLTT subgroups, based on the surgical approach to the hiatal ligament. Interestingly, a significant difference was observed in the local recurrence rate between the THLE and HLTT groups (5‐year: 0.95% vs. 7.62%, *p* = 0.017). Similarly, there was a significant difference in LRFS between the HLTT and THLE groups (*p* = 0.023; Figure 3G). In contrast, there was no significant difference between the two groups in OS and DFS (Figure S2). COX multivariate analysis of the posterior group revealed that CRM (HR, 8.501, *p* = 0.015), TNM stage III (HR, 14.989, *p* = 0.038), and HLTT (HR, 16.180, *p* = 0.026) were independent risk factors for LRFS (Table 3).

**TABLE 3:** Univariate and multivariate analysis of local recurrence‐free survival in posterior tumor location after propensity matching.

Variable	Univariate analysis		Multivariate analysis	
	HR (95% CI)	*p*	HR (95% CI)	*p*
Sex				
Female	1 (reference)			
Male	1.384 (0.331–5.793)	0.656		
Age (year)	1.013 (0.947–1.082)	0.710		
BMI (kg/m^2^)	1.025 (0.930–1.130)	0.617		
ASA score				
I/II	1 (reference)			
III	2.174 (0.267–17.676)	0.468		
Distance from anal verge (cm)	1.007 (0.490–2.072)	0.984		
Tumor size (cm)	0.957 (0.545–1.680)	0.877		
CEA, ng/mL, *n* (%)				
≤ 5	1 (reference)			
> 5	1.181 (0.238–5.854)	0.838		
CA19‐9, ng/mL, *n* (%)				
≤ 37	1 (reference)			
> 37	0.044 (0.000–3152.738)	0.583		
Neoadjuvant therapy				
No	1 (reference)			
Yes	2.670 (0.554–12.871)	0.221		
Postoperative complications				
No	1 (reference)			
Yes	1.228 (0.248–6.091)	0.802		
Histological differentiation				
Well/Moderate	1 (reference)			
Poor	4.494 (0.553–36.545)	0.160		
Pathologic TNM stage				
Stage I	1 (reference)		1 (reference)	
Stage II	3.097 (0.193–49.798)	0.425	4.361 (0.193–98.736)	0.355
Stage III	10.842 (1.299–90.536)	**0.028**	14.989 (1.165–192.92)	**0.038**
pCR	3.070 (0.191–49.365)	0.429	5.174 (0.226–118.486)	0.304
CRM				
Negative	1 (reference)		1 (reference)	
Positive	20.751 (4.168–103.325)	**< 0.001**	8.501 (1.503–48.092)	**0.015**
Adjuvant therapy				
No	1 (reference)			
Yes	0.831 (0.208–3.323)	0.793		
Treatment of hiatal ligament				
THLE	1 (reference)		1 (reference)	
HLTT	8.263 (1.033–66.068)	**0.046**	16.180 (1.40–186.93)	**0.026**

*Note:* Bold values indicate a *p* value < 0.05, which is statistically significant.

Abbreviations: AV, anal verge; CRM, circumferential resection margin (tumor ≤ 1 mm from the margin); HLTT, hiatal ligament traditional transection group; HR, hazard ratio; pCR, pathological complete response; THLE, total hiatal ligament excision.

Similarly, a subgroup analysis was performed for the non‐posterior group. Notably, there was no difference in LRFS between the HLTT and THLE groups (Figure 3H). There was no significant difference in the local recurrence rate between the HLTT group and THLE group in patients with non‐posterior tumors (5‐year: 3.33% vs. 8.89%, *p* = 0.121). TNM stage III (HR, 8.831, *p* = 0.044) were identified as independent risk factors for LRFS in the non‐posterior wall group (Table S4).

## 4 Discussion

This study exclusively included patients with ultra‐low rectal cancer undergoing laparoscopic surgery to investigate the impact of CTL on oncologic outcomes and explore the prognostic value of a new approach for resection of the hiatal ligament. Our results collectively demonstrated that CTL is not a prognostic risk factor for low rectal cancer. In the subgroup analysis of posterior wall tumors, traditional transection of hiatal ligaments was identified as an independent risk factor for local tumor recurrence.

Some studies suggest that posterior wall tumors have a lower positive rate of CRM than anterior wall tumors [16, 17, 18, 19]. This can be ascribed to tumors in the anterior wall being in close proximity to the genitourinary system and limited mesorectal separations [20]. Inherent limitations of surgical position and the angle and range of motion of laparoscopic instruments pose challenges to achieving a clear circumferential margin for anterior wall cancers. However, numerous studies have established that there is no significant difference in CRM between posterior and anterior wall tumors [4, 9, 10]. Herein, posterior and non‐posterior tumors had similar CRM‐positive rates. In addition, the CRM rate across the cohort was 2.1%, which is consistent with the findings of previous studies in Asian countries [21, 22]. Our observations suggest that CTL is not associated with postoperative CRM‐positive rates or local recurrence rates for low rectal cancer performed by the same group of skilled colorectal surgeons.

Herein, the 3‐year OS and DFS of patients with low and middle rectal cancer after laparoscopic TME surgery were 91.0% and 83.4%, respectively. The 3‐year OS and DFS ranges previously reported by RCTs were 86.7%–91.7% and 74.8%–79.2%, respectively, in line with our results [23, 24]. The 3‐year local recurrence rate was 4.23% in this study, which was consistent with the observation of previous studies (2.6%–5.7%) [23, 24]. It is worthwhile recognizing that earlier studies have reported high heterogeneity in CTL‐based oncological outcomes in rectal cancer patients. Indeed, the OS and DFS of patients with posterior tumors have been documented to be significantly higher than those with anterior tumors [4, 10]. In addition, Lee et al. [16] identified no significant difference in local recurrence rate between posterior and anterior tumors. However, a study undertaken by Franz et al. [17] showed that posterior tumors have a significantly lower local recurrence rate than anterior tumors. Furthermore, the results can vary depending on the ratio of chemoradiotherapy and the grouping criteria. In the current study, there was no significant difference in OS, DFS, or LRFS between posterior and non‐posterior wall tumors in the entire cohort. At the same time, the local recurrence rates were 4.58% and 6.33% in the posterior and non‐posterior groups, respectively. In multivariate analysis, only CRM status and pTNM stage III were identified as independent risk factors for OS, DFS, and LRFS of low rectal cancer.

The hiatal ligament is traditionally regarded as a ligamentous structure associated with the levator ani muscle rather than the rectum [25], which seals the hiatus of the levator ani muscle [26, 27]. Hence, its boundaries and total excision were conventionally deemed unimportant during hiatal ligament resection to access the intersphincteric space [28]. However, recent histological studies concluded that the hiatal ligament is a continuation of the longitudinal muscle of the rectum that contains blood vessels and lymphatic vessels [6, 27]. Consequently, ultra‐low rectal cancer in the posterior wall may directly spread or metastasize through the hiatal ligament. From an embryological perspective, the hiatal ligament lies within the boundaries of the TME. In summary, the traditional approach to resecting the hiatal ligament does not emphasize the exposure of its boundaries and fails to achieve total excision of the hiatal ligament. Histologically, the residual hiatal ligament represents residual rectal wall tissue, which may contribute to local tumor recurrence. Therefore, in the context of very low rectal cancer located in the posterior wall, ultra‐low anterior resection (ULAR) should aim to identify and comprehensively excise the hiatal ligament.

Across the cohort, no significant association was noted between traditional transection of hiatal ligaments and prognosis in low rectal cancer. Following this, the posterior group was further subdivided into the HLTT and THLE subgroups, exposing a significant difference in local recurrence rates between the two subgroups: 7.62% in the HLTT subgroup and 0.95% in the THLE subgroup. However, in the non‐posterior group, THLE had no significant effect on local recurrence. Besides, CRM status, pTNM stage III, and transection of the hiatal ligament were identified as independent risk factors for LRFS in the multivariate analysis of the posterior group.

In addition, there are several clinical risk factors affecting postoperative local control of low and middle rectal cancer, such as age, gender, narrow pelvic cavity, bulky mesentery, etc. Noteworthily, a series of recent studies pointed out that robotic techniques significantly influence local recurrence rates in mid/low rectal cancer patients, especially among males, compared to the laparoscopic group [29, 30]. Male patients often face challenges such as a narrow pelvic cavity, bulky mesentery, and irradiated pelvis, which can be effectively overcome by robotic tools [30]. Interestingly, in female patients with rectal cancer who performed TME, no significant differences were noted in oncologic outcomes between robotic (rob) and laparoscopic (lap) approaches [31]. However, the robotic surgical platform incurs a higher cost compared to laparoscopy, thereby limiting its accessibility. The implementation of robotic surgery is often tailored and prioritized in resource‐constrained healthcare centers. The absence of cases undergoing surgery using robotic approaches in this study restricted our ability to comprehensively evaluate the potential benefits of robotic surgery in this specific patient population.

Nevertheless, some limitations of this study warrant acknowledgment. To begin, the retrospective nature of this study may have introduced selection bias. Secondly, future prospective multicenter studies are warranted to validate our findings. To our knowledge, this is the first study to demonstrate the impact of different approaches for the resection of the hiatal ligament on the local recurrence rate following surgery for ultra‐low rectal cancer.

## 5 Conclusions

Our study shows that CTL is not an independent risk factor for OS, DFS, and local recurrence after laparoscopic TME surgery for low rectal cancer. Moreover, THLE significantly reduces the risk of local recurrence after laparoscopic TME for ultra‐low rectal cancer located in the posterior wall.

## Author Contributions

**Shoufeng Li:** conceptualization (supporting), data curation (equal), formal analysis (equal), funding acquisition (equal), software (equal). **Ye Wang:** conceptualization (supporting), data curation (equal), formal analysis (equal). **Huajun Cai:** conceptualization (supporting), validation (equal). **Zhen Pan:** conceptualization (supporting), resources (equal). **Xing Liu:** conceptualization (supporting), supervision (equal). **Jinfu Zhuang:** conceptualization (supporting). **Guoxian Guan:** conceptualization (lead), data curation (equal), formal analysis (equal), funding acquisition (equal), investigation (equal), methodology (equal), project administration (equal), resources (lead), software (equal), supervision (equal), validation (equal), visualization (equal), writing – original draft (equal), writing – review and editing (supporting).

## Ethics Statement

This study was approved by the Institutional Review Board (IRB) of The First Affiliated Hospital of Fujian Medical University. We confirm that the study was conducted in accordance with the relevant guidelines/regulations, and all participants and/or their legal guardians provided informed consent.

## Consent

Informed consent for research purpose with patient data and images were obtained for all patients.

## Conflicts of Interest

The authors declare no conflicts of interest.

## Supporting information


**Figure S1.** Circumferential tumor location grouping. (A). The rectal wall was divided into 3 sections, namely anterior, lateral, and posterior. (B, C). The posterior quadrant is considered the back position and corresponds to the 4:00 to 8:00 o ‘clock position. If a tumor involves the posterior quadrant, it is categorized as a posterior quadrant tumor regardless of involvement in other quadrants (excluding circumferential tumors). (D). Tumors that do not involve the posterior part are considered non‐posterior wall tumors.

**Figure S2.** Kaplan–Meier curves displaying overall survival and disease‐free survival in posterior wall tumors in the propensity score matching‐adjusted cohort.

Table S1.

Table S2.

Table S3.

Table S4.

## Data Availability

Some or all data used during the study are available from the corresponding author by request.
