# Supplementary figures and images for: A New Approach to Ultra‐Low Anterior Resection—Intersphincteric Dissection With Total Hiatal Ligament Excision for Very Low Rectal Cancer Located in the Posterior Wall of the Rectum: A More Satisfactory Technique for Local Recurrence Control
Source: Cancer Med. 2024 Oct 10;13(19):e70307. doi: 10.1002/cam4.70307 (PMC11465284; doi:10.1002/cam4.70307)

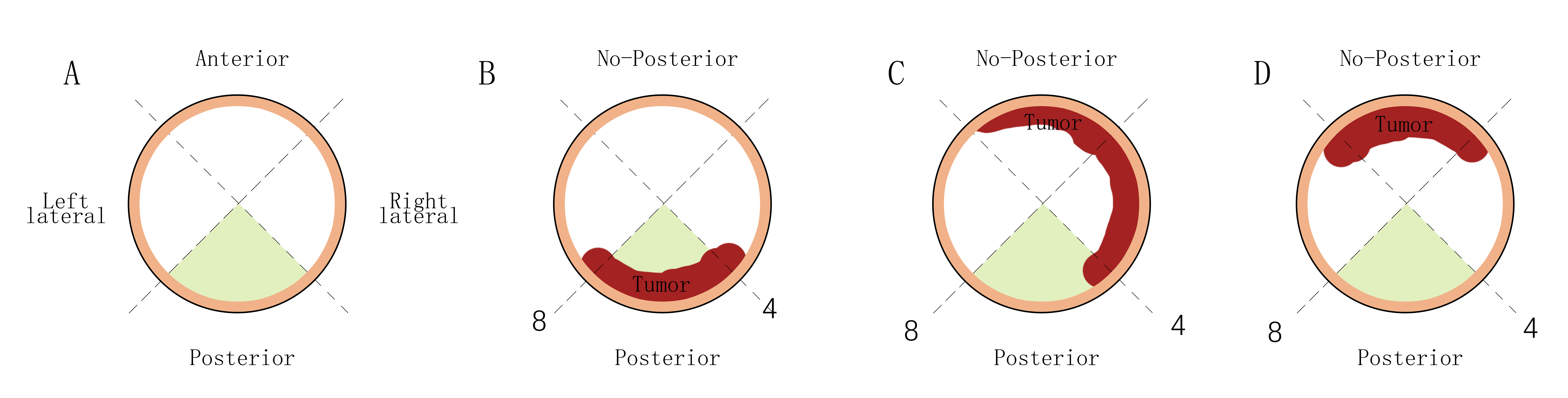

Supplement: Supplementary file 1 — Figure S1. Circumferential tumor location grouping. (A). The rectal wall was divided into 3 sections, namely anterior, lateral, and posterior. (B, C). The posterior quadrant is considered the back position and corresponds to the 4:00 to 8:00 o ‘clock position. If a tumor involves the posterior quadrant, it is categorized as a posterior quadrant tumor regardless of involvement in other quadrants (excluding circumferential tumors). (D). Tumors that do not involve the posterior part are considered non‐posterior wall tumors. [file CAM4-13-e70307-s006.tif]

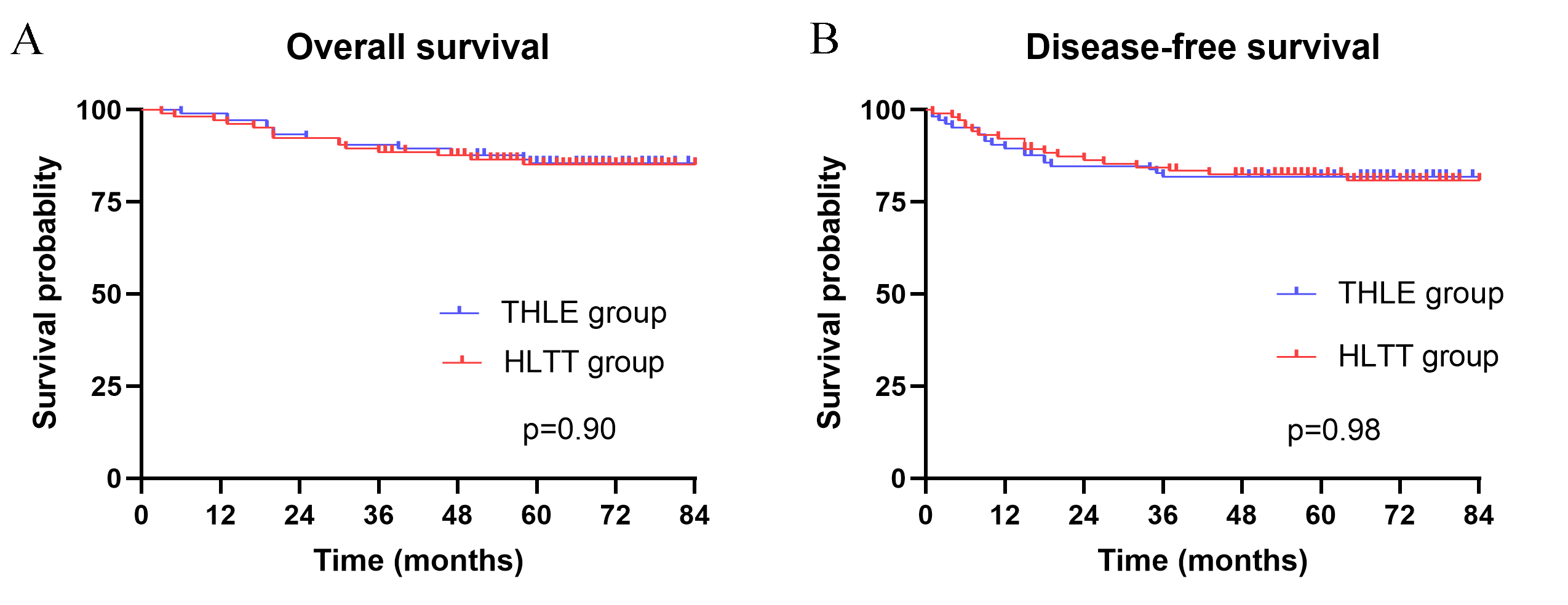

Supplement: Supplementary file 2 — Figure S2. Kaplan–Meier curves displaying overall survival and disease‐free survival in posterior wall tumors in the propensity score matching‐adjusted cohort. [file CAM4-13-e70307-s005.tif]
