# Supplementary material for: A New Approach to Ultra‐Low Anterior Resection—Intersphincteric Dissection With Total Hiatal Ligament Excision for Very Low Rectal Cancer Located in the Posterior Wall of the Rectum: A More Satisfactory Technique for Local Recurrence Control
Source: Cancer Med. 2024 Oct 10;13(19):e70307. doi: 10.1002/cam4.70307 (PMC11465284; doi:10.1002/cam4.70307)
Supplement: Supplementary file 3 — Table S1. [file CAM4-13-e70307-s001.docx]

**Supplementary Table 1.** Univariate analysis of overall survival and disease-free survival in the post-matching cohort

| Variable | Overall survival | | Disease-free survival | |
| --- | --- | --- | --- | --- |
|  | HR (95% CI) | p value | HR (95% CI) | p value |
| Sex |  |  |  |  |
| Female | 1 (reference) |  | 1 (reference) |  |
| Male | 1.368 (0.736-2.542) | 0.322 | 0.975 (0.606-1.569) | 0.917 |
| Age (year) | 0.984 (0.956-1.012) | 0.259 | 1.002 (0.978-1.025) | 0.895 |
| BMI (kg/m2) | 0.978 (0.899-1.064) | 0.605 | 0.980 (0.916-1.048) | 0.549 |
| ASA score |  |  |  |  |
| I/II | 1 (reference) |  | 1 (reference) |  |
| III | 0.525 (0.127-2.169) | 0.374 | 0.713 (0.260-1.956) | 0.713 |
| Distance from anal verge (cm) | 0.985 (0.689-1.408) | 0.932 | 1.033 (0.778-1.372) | 0.820 |
| Tumor size (cm) | 1.032 (0.793-1.343) | 0.817 | 1.060 (0.861-1.306) | 0.582 |
| CEA, ng/mL, n (%) |  |  |  |  |
| ≤5 | 1 (reference) |  | 1 (reference) |  |
| ＞5 | 0.969 (0.467-2.011) | 0.932 | 1.896 (1.144-3.141) | **0.013** |
| CA19-9, ng/mL, n (%) |  |  |  |  |
| ≤37 | 1 (reference) |  | 1 (reference) |  |
| ＞37 | 1.355 (0.535-3.434) | 0.522 | 0.835 (0.336-2.073) | 0.697 |
| Neoadjuvant therapy |  |  |  |  |
| [No](javascript:;) | 1 (reference) |  | 1 (reference) |  |
| Yes | 0.822 (0.431-1.566) | 0.552 | 1.142 (0.685-1.904) | 0.611 |
| Postoperative complications |  |  |  |  |
| [No](javascript:;) | 1 (reference) |  | 1 (reference) |  |
| Yes | 1.031 (0.532-1.997) | 0.928 | 1.052 (0.621-1.782) | 0.850 |
| Histological differentiation |  |  |  |  |
| Well / Morderate | 1 (reference) |  | 1 (reference) |  |
| Poor | 20.990 (0.009-61441.2) | 0.445 | 20.952 (0.007-60551.3) | 0.514 |
| Pathologic TNM stage |  |  |  |  |
| Stage I | 1 (reference) |  | 1 (reference) |  |
| Stage II | 2.075 (0.824-5.229) | 0.121 | 1.138 (0.525-2.465) | 0.744 |
| Stage III | 3.765 (1.742-8.138) | **0.001** | 3.091 (1.760-5.431) | **<0.001** |
| pCR | 1.096 (0.337-3.558) | 0.879 | 0.636 (0.236-1.713) | 0.371 |
| CRM |  |  |  |  |
| Negative | 1 (reference) |  | 1 (reference) |  |
| Positive | 7.931 (3.351-18.768) | **<0.001** | 6.126 (2.800-13.406) | **<0.001** |
| Adjuvant therapy |  |  |  |  |
| No | 1 (reference) |  | 1 (reference) |  |
| Yes | 1.036 (0.525-2.0446) | 0.918 | 1.099 (0.649-1.861) | 0.726 |
| Circumferential involvement |  |  |  |  |
| No-posterior | 1 (reference) |  | 1 (reference) |  |
| Posterior | 1.031 (0.575-1.850) | 0.918 | 1.102 (0.689-1.762) | 0.685 |
| Treatment of hiatal ligament |  |  |  |  |
| THLE | 1 (reference) |  | 1 (reference) |  |
| HLTT | 1.216 (0.677-2.186) | 0.513 | 1.105 (0.690-1.770) | 0.676 |

Abbreviations: AV, anal verge; CRM, circumferential resection margin (tumour ≤1 mm from the margin); HR, hazard ratio; pCR, pathological complete response; THLE, total hiatal ligament excision; HLTT, hiatal ligament traditional transection group
