# Supplementary material for: A New Approach to Ultra‐Low Anterior Resection—Intersphincteric Dissection With Total Hiatal Ligament Excision for Very Low Rectal Cancer Located in the Posterior Wall of the Rectum: A More Satisfactory Technique for Local Recurrence Control
Source: Cancer Med. 2024 Oct 10;13(19):e70307. doi: 10.1002/cam4.70307 (PMC11465284; doi:10.1002/cam4.70307)
Supplement: Supplementary file 4 — Table S2. [file CAM4-13-e70307-s003.docx]

**Supplementary Table 2.** Univariate analysis of local recurrence-free survival in the post-matching cohort

| Variable | Local recurrence-free survival | |
| --- | --- | --- |
|  | HR (95% CI) | p value |
| Sex |  |  |
| Female | 1 (reference) |  |
| Male | 1.082 (0.441-2.656) | 0.864 |
| Age (year) | 0.981 (0.940-1.023) | 0.367 |
| BMI (kg/m2) | 0.993 (0.885-1.115) | 0.910 |
| ASA score |  |  |
| I/II | 1 (reference) |  |
| III | 1.312 (0.304-5.663) | 0.716 |
| Distance from anal verge (cm) | 1.042 (0.611-1.776) | 0.880 |
| Tumor size (cm) | 0.931 (0.622-1.395) | 0.730 |
| CEA, ng/mL, n (%) |  |  |
| ≤5 | 1 (reference) |  |
| ＞5 | 2.727 (1.108-6.706) | **0.029** |
| CA19-9, ng/mL, n (%) |  |  |
| ≤37 | 1 (reference) |  |
| ＞37 | 0.540 (0.072-4.050) | 0.549 |
| Neoadjuvant therapy |  |  |
| [No](javascript:;) | 1 (reference) |  |
| Yes | 4.560 (1.057-19.669) | **0.042** |
| Postoperative complications |  |  |
| [No](javascript:;) | 1 (reference) |  |
| Yes | 2.027 (0.593-6.923) | 0.260 |
| Histological differentiation |  |  |
| Well / Morderate | 1 (reference) |  |
| Poor | 1.371 (0.169-11.123) | 0.768 |
| Pathologic TNM stage |  |  |
| Stage I | 1 (reference) |  |
| Stage II | 2.066 (0.291-14.674) | 0.468 |
| Stage III | 10.420 (2.366-45.885) | **0.002** |
| pCR | 2.425 (0.342-17.223) | 0.376 |
| CRM |  |  |
| Negative | 1 (reference) |  |
| Positive | 11.030 (3.193-38.099) | **<0.001** |
| Adjuvant therapy |  |  |
| No | 1 (reference) |  |
| Yes | 1.896 (0.555-6.477) | 0.307 |
| Circumferential involvement |  |  |
| No-posterior | 1 (reference) |  |
| Posterior | 0.651 (0.266-1.593) | 0.347 |
| Treatment of hiatal ligament |  |  |
| THLE | 1 (reference) |  |
| HLTT | 1.461 (0.604-3.531) | 0.400 |

Abbreviations: SD, standard deviation; CRM, circumferential resection margin (tumour ≤1 mm from the margin); pCR, pathological complete response; THLE, total hiatal ligament excision; HLTT, hiatal ligament traditional transection group
