# Supplementary material for: A New Approach to Ultra‐Low Anterior Resection—Intersphincteric Dissection With Total Hiatal Ligament Excision for Very Low Rectal Cancer Located in the Posterior Wall of the Rectum: A More Satisfactory Technique for Local Recurrence Control
Source: Cancer Med. 2024 Oct 10;13(19):e70307. doi: 10.1002/cam4.70307 (PMC11465284; doi:10.1002/cam4.70307)
Supplement: Supplementary file 5 — Table S3. [file CAM4-13-e70307-s004.docx]

**Supplementary Table 3.** Patient characteristics before and after propensity score matching in posterior tumor location

| Variable | Unmatched cohort | |  | PSM-adjusted cohort | |  |
| --- | --- | --- | --- | --- | --- | --- |
|  | HLTT (n=107) | THLE (n=133) | P value | HLTT  (n=105) | THLE (n=105) | P value |
| Sex, n (%) |  |  | 0.585 |  |  | 0.677 |
| Male | 59 (55.1) | 78 (58.6) |  | 59 (56.2) | 56 (53.3) |  |
| Female | 48 (44.9) | 55 (41.4) |  | 46 (43.8) | 49 (46.7) |  |
| Age (year) Mean± SD | 57.3±10.6 | 52.7±11.9 | **0.002** | 55.6±10.6 | 55.5±10.5 | 0.969 |
| BMI (kg/m2) ± SD | 23.46±6.3 | 22.4±2.8 | 0.095 | 22.9±6.3 | 22.3±2.6 | 0.340 |
| ASA score, n (%) |  |  | 0.491 |  |  | 0.390 |
| I/II | 102 (95.3) | 124 (93.2) |  | 100 (95.2) | 97 (92.4) |  |
| III | 5 (4.7) | 9 (6.8) |  | 5 (4.8) | 8 (7.6) |  |
| Distance from anal verge (cm), mean ± SD | 3.9±0.9 | 3.8±0.9 | 0.198 | 3.9±0.8 | 3.8±0.9 | 0.265 |
| Tumor size (cm), mean ± SD | 2.8±1.3 | 2.6±1.2 | 0.318 | 2.7±1.2 | 2.6±1.2 | 0.62 |
| CEA, ng/mL, n (%) |  |  | 0.872 |  |  | 0.898 |
| ≤5 | 83 (77.6) | 102 (76.7) |  | 81 (77.1) | 82 (78.1) |  |
| ＞5 | 24 (22.4) | 31 (23.3) |  | 24 (22.9) | 23 (21.9) |  |
| CA19-9, ng/mL, n (%) |  |  | 0.181 |  |  | 0.448 |
| ≤37 | 99 (92.5) | 116 (87.2) |  | 98 (93.3) | 95 (90.5) |  |
| ＞37 | 8 (17.5) | 17 (12.8) |  | 7 (6.7) | 10 (9.5) |  |
| Neoadjuvant therapy, n (%) |  |  | 0.057 |  |  | 0.370 |
| Short-course radiotherapy | 0 (0.0) | 2 (1.5) |  | 0 (0.0) | 2 (1.9) |  |
| [Chemotherapy](javascript:;) | 0 (0.0) | 1 (0.8) |  | 0 (0.0) | 1 (1.0) |  |
| Chemoradiotherapy | 61 (57.0) | 83 (62.4) |  | 61 (58.1) | 57 (54.3) |  |
| None | 46 (43.0) | 47 (35.3) |  | 44 (41.9) | 45 (42.9) |  |
| Operative time, min, mean ± SD | 203.3±48.9 | 215.2±48.5 | 0.062 | 269.8±47.2 | 210.3±48.3 | 0.590 |
| Blood loss, mL, median (range) | 50 (10-400) | 50 (5-400) | 0.465 | 50 (10-400) | 50 (10-200) | 0.767 |
| Postoperative complications, n (%) | 31 (28.9) | 28 (21.1) | 0.82 | 26 (24.8) | 22 (21.0) | 0.511 |
| Distal cutting margin (cm), mean ± SD | 1.9±0.6 | 1.8±0.5 | 0.508 | 1.8±0.6 | 1.8±0.5 | 0.897 |
| Pathologic T stage, n (%) |  |  | 0.319 |  |  | 0.276 |
| pT1 | 9 (8.4) | 17 (12.8) |  | 9 (8.6) | 17 (13.3) |  |
| pT2 | 34 (31.8) | 51 (38.3) |  | 34 (32.4) | 27 (40.0) |  |
| pT3 | 40 (37.4) | 37 (27.8) |  | 39 (37.1) | 42 (25.7) |  |
| pT4 | 1 (0.9) | 0 (0.0) |  | 1 (1.0) | 0 (0.0) |  |
| pT0 | 23 (21.5) | 28 (21.1) |  | 22 (21.0) | 22 (20.7) |  |
| Pathologic N stage, n (%) |  |  | 0.249 |  |  | 0.606 |
| pN0 | 76 (71.0) | 95 (71.4) |  | 75 (71.4) | 75 (71.4) |  |
| pN1 | 23 (21.5) | 34 (25.6) |  | 23 (21.9) | 26 (24.8) |  |
| pN2 | 8 (7.5) | 4 (3.0) |  | 7 (6.7) | 4 (3.8) |  |
| Pathologic TNM stage, n (%) |  |  | 0.751 |  |  | 0.253 |
| I | 41 (38.3) | 58 (43.6) |  | 41 (39.0) | 51 (48.6) |  |
| II | 22 (20.6) | 22 (16.5) |  | 22 (21.0) | 12 (11.4) |  |
| III | 26 (24.3) | 34 (25.6) |  | 25 (23.8) | 26 (24.8) |  |
| pCR | 18 (16.8) | 19 (14.3) |  | 17 (16.2) | 16 (15.7) |  |
| Histological differentiation, n (%) |  |  | 0.545 |  |  | 0.305 |
| Well | 2 (1.9) | 1 (0.8) |  | 2 (1.9) | 0 (0.0) |  |
| Moderate | 103 (96.3) | 131 (98.5) |  | 101 (96.2) | 104 (99.0) |  |
| Poor | 2 (1.9) | 1 (0.8) |  | 1 (1.9) | 1 (0.0) |  |
| CRM, n (%) |  |  | 0.835 |  |  | 1.000 |
| Negative (>1 mm) | 105 (98.1) | 130 (97.7) |  | 103 (98.1) | 103 (98.1) |  |
| Positive (≤1 mm) | 2 (1.9) | 3 (2.3) |  | 2 (1.9) | 2 (1.9) |  |
| Adjuvant therapy, n |  |  | 0.262 |  |  | 0.210 |
| [Chemotherapy](javascript:;) | 76 (71.0) | 96 (72.2) |  | 75 (71.4) | 71 (67.6) |  |
| Chemoradiotherapy | 0 (0.0) | 3 (2.3) |  | 0 (0.0) | 3 (2.9) |  |
| None | 31 (29.0) | 34 (25.6) |  | 30 (28.6) | 31 (29.5) |  |

Abbreviations: SD, standard deviation; CRM, circumferential resection margin (tumour ≤1 mm from the margin); pCR, pathological complete response; THLE, total hiatal ligament excision; HLTT, hiatal ligament traditional transection group
