# Supplementary material for: A New Approach to Ultra‐Low Anterior Resection—Intersphincteric Dissection With Total Hiatal Ligament Excision for Very Low Rectal Cancer Located in the Posterior Wall of the Rectum: A More Satisfactory Technique for Local Recurrence Control
Source: Cancer Med. 2024 Oct 10;13(19):e70307. doi: 10.1002/cam4.70307 (PMC11465284; doi:10.1002/cam4.70307)
Supplement: Supplementary file 6 — Table S4. [file CAM4-13-e70307-s002.docx]

**Supplementary Table 4.** Univariate and Multivariate analysis of local recurrence-free survival in no-posterior tumor location

| Variable | Univariate analysis | | Multivariate analysis | |
| --- | --- | --- | --- | --- |
|  | HR (95% CI) | p value | HR (95% CI) | p value |
| Sex |  |  |  |  |
| Female | 1 (reference) |  |  |  |
| Male | 0.982 (0.277-3.479) | 0.977 |  |  |
| Age (year) | 0.999 (0.935-1.067) | 0.969 |  |  |
| BMI (kg/m2) | 0.876 (0.725-1.058) | 0.169 |  |  |
| ASA score |  |  |  |  |
| I | 1 (reference) |  |  |  |
| II | 0.300 (0.079-1.149) | 0.079 |  |  |
| III | 0.533 (0.061-4.699) | 0.571 |  |  |
| Distance from anal verge (cm) | 0.872 (0.359-2.120) | 0.762 |  |  |
| Tumor size (cm) | 0.714 (0.347-1.469) | 0.360 |  |  |
| CEA, ng/mL, n (%) |  |  |  |  |
| ≤5 | 1 (reference) |  |  |  |
| ＞5 | 3.118 (0.837-11.616) | 0.090 |  |  |
| CA19-9, ng/mL, n (%) |  |  |  |  |
| ≤37 | 1 (reference) |  |  |  |
| ＞37 | 0.046 (0.000-21212.205) | 0.643 |  |  |
| Neoadjuvant therapy |  |  |  |  |
| [No](javascript:;) | 1 (reference) |  |  |  |
| Yes | 5.652 (0.623-51.261) | 0.124 |  |  |
| Postoperative complications |  |  |  |  |
| [No](javascript:;) | 1 (reference) |  |  |  |
| Yes | 1.759 (0.360-8.605) | 0.486 |  |  |
| Histological differentiation |  |  |  |  |
| Well / Morderate | 1 (reference) |  |  |  |
| Poor | 24.549 (0.000-97049907) | 0.680 |  |  |
| Pathologic TNM stage |  |  |  |  |
| Stage I | 1 (reference) |  | 1 (reference) |  |
| Stage II | 2.230 (0.139-35.662) | 0.571 | 2.228 (0.139-35.621) | 0.571 |
| Stage III | 9.965 (1.224-81.137) | **0.032** | 8.831 (1.061-73.528) | **0.044** |
| pCR | 2.390 (0.149-38.421) | 0.539 | 2.416 (0.150-38.883) | 0.534 |
| CRM |  |  |  |  |
| Negative | 1 (reference) |  | 1 (reference) |  |
| Positive | 11.493 (1.411-93.612) | **0.023** | 4.517 (0.524-38.905) | 0.170 |
| Adjuvant therapy |  |  |  |  |
| No | 1 (reference) |  |  |  |
| Yes | 1.288 (0.273-6.071) | 0.749 |  |  |
| Treatment of hiatal ligament |  |  |  |  |
| THLE | 1 (reference) |  |  |  |
| HLTT | 1.915 (0.479-7.663) | 0.358 |  |  |

Abbreviations: AV, anal verge; CRM, circumferential resection margin (tumour ≤1 mm from the margin); HR, hazard ratio; pCR, pathological complete response; THLE, total hiatal ligament excision; HLTT, hiatal ligament traditional transection group
